# cGAS–STING and MyD88 Pathways Synergize in Ly6C^hi^ Monocyte to Promote *Streptococcus pneumoniae*-Induced Late-Stage Lung IFNγ Production

**DOI:** 10.3389/fimmu.2021.699702

**Published:** 2021-08-26

**Authors:** Seema Patel, Heidi R. Tucker, Himanshu Gogoi, Samira Mansouri, Lei Jin

**Affiliations:** ^1^Division of Pulmonary, Critical Care and Sleep Medicine, Department of Medicine, University of Florida, Gainesville, FL, United States; ^2^Department of Immunology and Microbial Disease, Albany Medical College, Albany, NY, United States

**Keywords:** STING, IFNγ, monocyte, MyD88, *Streptococcus pneumoniae (pneumococcus)*

## Abstract

The cyclic GMP–AMP synthase–stimulator of interferon genes (cGAS–STING) pathway senses DNA and induces type I interferon (IFN) production. Whether and how the STING pathway crosstalk to other innate immune pathways during pathogen infection, however, remains unclear. Here, we showed that STING was needed for *Streptococcus pneumoniae*-induced late, not early, stage of lung IFNγ production. Using knockout mice, IFNγ reporter mice, intracellular cytokine staining, and adoptive cell transfer, we showed that cGAS–STING-dependent lung IFNγ production was independent of type I IFNs. Furthermore, STING expression in monocyte/monocyte-derived cells governed IFNγ production in the lung *via* the production of IL-12p70. Surprisingly, DNA stimulation alone could not induce IL-12p70 or IFNγ in Ly6C^hi^ monocyte. The production of IFNγ required the activation by both DNA and heat-killed *S. pneumococcus*. Accordingly, MyD88^−/−^ monocyte did not generate IL-12p70 or IFNγ. In summary, the cGAS–STING pathway synergizes with the MyD88 pathway in monocyte to promote late-stage lung IFNγ production during pulmonary pneumococcal infection.

## Introduction

During pathogen infections, multiple innate immune signaling pathways are activated. Stimulator of interferon genes (STING) is essential for cytosolic DNA-induced type I interferon (IFN) production but largely dispensable for Toll-like receptors (TLRs) activations (1–3). Currently, it is not clear if and how the STING pathway crosstalks with another innate immune pathway during infections.

*Streptococcus pneumoniae* is an extracellular bacterial pathogen that causes pneumonia, sinusitis, otitis media, septicemia, and meningitis (4, 5). A recent study found that the STING-mediated cytosolic DNA sensing pathway is activated during pulmonary *S. pneumoniae* infection (6). However, pneumococcal infection-induced proinflammatory cytokines, including tumor necrosis factor α (TNFα), interleukin (IL)-6, and IL-1β, are largely intact in the STING^−/−^ mice, and the bacterial burden in the lung, spleen, and blood were comparable between STING^−/−^ and wild-type (WT) mice (6). Thus, STING seems to be dispensable for the initial innate immunity to *S. pneumoniae* including the control of bacterial burden.

IFNγ promotes M1-macrophage development that not only phagocyte and kill the bacteria but also contribute to tissue injury. *Streptococcus pneumoniae* infection induces lung IFNγ. In patients with *S. pneumoniae* sepsis, plasma IFNγ was elevated and correlated with increased mortality (7). IFNγ^−/−^ mice are more resistant than the WT mice in developing pneumococcal meningitis (8). For pneumococcal pneumonia, IFNγ^−/−^ mice or pretreatment of mice with anti-IFNγ neutralizing Ab had either no effect on mortality (9, 10) or lead to decreased survival (11, 12). Thus, the role of lung IFNγ production during pulmonary pneumococcal infection remains controversial.

In this report, we found that there were two waves of lung IFNγ productions by different immune cells during pulmonary pneumococcal infection. STING is required for the late, not early, stage of lung IFNγ production. Notably, the production of IFNγ required the activation of both STING and MyD88 pathways indicating previous unknown crosstalk between STING and TLRs pathways during infection.

## Materials and Methods

### Mice

Mice 8- to 16 weeks old, both males and females, were used for all experiments. STING^−/−^ mice (*tmem173*<tm1Camb>) have been described previously (13). The STING^flox/flox^/*TMEM173* ^flox/flox^ mouse has been described previously (14). The following strains were obtained from The Jackson Laboratory: CCR2^−/−^, cyclic GMP–AMP synthase (cGAS)^−/−^, IL-12p70^−/−^, IFNRA1^−/−^, MyD88^−/−^, TLR2^−/−^, and IFNγ YFP-reporter.

All mice are on a C57BL/6 background. Mice were housed and bred in the Animal Research Facility at the University of Florida. All experiments with mice were performed by the regulations and approval of the Institutional Animal Care and Use Committee from the University of Florida (protocol number 201909362).

### *Streptococcus pneumoniae* Infection

*Streptococcus pneumoniae* D39 (serotype 2) were grown in tryptic soy broth (TSB) at 37°C to an optical density (OD) of 0.45–0.50 at 600 nm (∼10^8^ CFU/ml). Mice were intranasally administered ∼8–10 × 10^6^ CFU in 50 µl of 1× Ultrapure phosphate-buffered saline (PBS). CFUs were confirmed by colony counting of log_10_ serial dilutions of bacteria cultured overnight on a TSB with a 10% sheep blood agar plate.

### BacLight Green Stained *Streptococcus pneumoniae* Infection

*Streptococcus pneumoniae* D39 (serotype 2) were grown in TSB at 37°C to an OD of 0.35–0.4 at 600 nm; BacLight green (1 µg/ml) was added and incubated at 37°C to an OD of 0.45–0.5 at 600 nm (∼10^8^ CFU/ml). Mice were intranasally administered with ∼8–10 × 10^6^ CFU.

### Detection of the Lung Cytokine Production

Mice were intranasally infected with *S. pneumoniae* D39. Mice were sacrificed by CO_2_ asphyxiation at the indicated time points. The lungs were subsequently perfused with cold PBS, washed in PBS once, and stored in a 1.0-ml tissue protein extraction reagent (T-PER) containing protease inhibitors (Roche, Indianapolis, IN). The lungs were homogenized using a Bertin Technology Minilys tissue homogenizer. Lung homogenates were spun at 14,000×*g* for 15 min at 4°C. The supernatant was collected and analyzed for cytokine production using ELISA.

### Cytokine ELISAs

Cytokine concentrations were measured using ELISA kits from eBiosciences according to the manufacturer’s instructions. The ELISA kits used were IL-1β, IL-6, IL-12/p70, TNFα, monocyte chemoattractant protein-1 (MCP-1), and IFNγ. The IFNβ ELISA kit was from PBI Interferon Source, Piscataway, NJ.

### Flow Cytometry Analysis

Mice were intranasally infected with *S. pneumoniae* D39. Mice were sacrificed by CO_2_ asphyxiation at the indicated time points. The lungs were subsequently perfused with cold PBS. Excised lungs were cut into small pieces and digested in Roswell Park Memorial Institute (RPMI) containing 200 μg/ml DNase I (Roche) and 25 μg/ml Liberase TM (Roche) at 37°C for 2 h. Red blood cells were then lysed using ACK lysis buffer (Gibco), and a single-cell suspension was prepared and analyzed by BD LSR Fortessa flow cytometry.

The following Abs from Biolegend were used in the flow cytometry: Ly6C (HK1.4), CD11b (M1/70), Ly6G (1A8), CD11c (N418), NK-1.1 (PK136), MHC II (M5/114.15.2), CD103 (2E7), CD3 (145.2C11), CD64 (X54-5/7.1), Siglec F (S17007L), IFNγ (XMG1.2), and IL-12p70 (27537).

### Intracellular Staining

The intracellular cytokine staining was performed using the Cytofix/Cytoperm™ kit from BD Biosciences. Briefly, mice were intranasally administered with either *S. pneumoniae* D39 ∼8–10 × 10^6^ CFU in 50 µl of 1× Ultrapure PBS or PBS alone. The lungs were perfused and harvested at 24 and 48 h postinfection and washed in PBS followed by 2 h of digestion in RPMI containing 200 µg/ml DNAse I (Roche), 25 µg/ml Librase TM (Roche), and Golgi-plug 1 µg/µl (BD Bioscience). Digested lungs were processed to prepare the single lung cell suspension in RPMI containing Golgi-plug 1 µg/µl. The cells were fixed in Cytofix/perm buffer (BD Biosciences) in the dark for 20 min at room temperature (RT). Fixed cells were washed and kept in Perm/Wash buffer at 4°C. The Golgi-plug was present during every step before fixation. Cells were stained with cytokine-specific staining antibodies in perm buffer in the dark for 30 min at RT. Cells were washed, and the single-cell suspension was prepared to be analyzed by BD LSRFortessa flow cytometry.

### *Ex Vivo* Monocyte Culture and Activation

Ly6C^hi^ monocytes were purified from bone marrow cells using EasySep Mouse Monocyte Isolation kit (STEMCELL Technologies). Purified monocytes were cultured in RPMI (Invitrogen) with 10% FBS, 2 mM L-glutamine, 1 mM sodium pyruvate, 10 mM HEPES buffer, 1% non-essential amino acids, 50 μM 2-mercaptoethanol, and1% Pen/Strep. Cells were stimulated with 5 × 10^6^ CFU/ml heat-killed *S. pneumoniae* (HKSP) (InvivoGen), 2 µg/ml apoptotic DNA transfected with lipofectamine 3000 (15), or both for 17 h at 37°C. To generate apoptotic DNA, we isolated splenocytes from WT mice and cultured those in complete RPMI for 3 days at 37°C without changing the media; cells were used to isolate genomic DNA using a Qiagen kit. The supernatant of stimulated Ly6C^hi^ monocytes was analyzed for cytokine production.

### Statistical Analysis

All data were expressed as means ± SEM. Statistical significance was evaluated using Prism 9.1 software to perform one-way ANOVA Tukey’s multiple comparison test.

## Results

### *Streptococcus pneumoniae*-Induced Innate Immune Responses Are Largely Intact in STING^−/−^ Mice

We investigated the role of STING in host defense against pulmonary *S. pneumoniae* infection. *Streptococcus pneumoniae*-induced lung inflammatory cytokines IL-6, IL-1β, TNFα, and chemokines KC, MCP-1 were not altered in the STING^−/−^ mice (**Figures S1A–E**). Interestingly, there was no detectable *S. pneumoniae*-induced lung IFNβ protein (**Figure S1F**). Lung bacterial burden and total proteins in the BAL fluid, an indication of lung damage, were also not significantly different between STING^−/−^ and WT mice (**Figures S1G, H**). Pneumococcal infection recruits neutrophils into the lung that are critical for the host defense (16). There was no difference in the total numbers of recruited neutrophils (CD11b^hi^Ly6G^+^) between WT and STING^−/−^ mice (**Figures S1I, J**). Thus, STING is largely dispensable for the innate immune responses to pulmonary pneumococcal infection, which is consistent with a recent report (6).

### STING Is Required for *S. pneumoniae* Induced Lung IL-12p70 by Monocyte/Monocyte-Derived Cells

**Figure 1:**
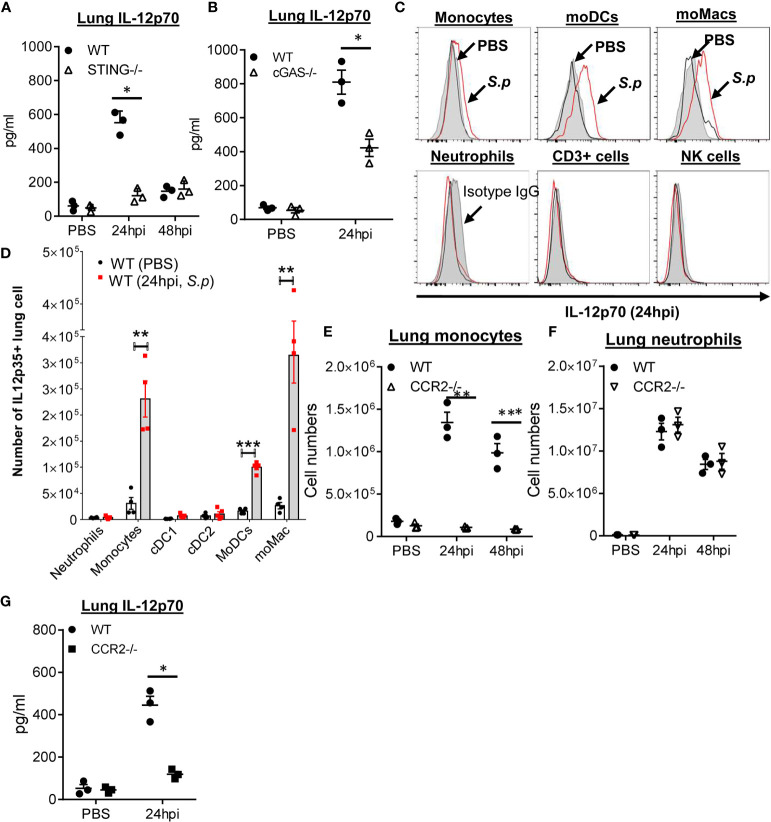
cGAS–STING mediate *S. pneumoniae*-induced lung IL-12p70 production by monocyte/monocyte-derived cells. **(A, B)** STING^−/−^, cGAS^−/−^, and WT littermates mice were given PBS or infected (i.n.) with *S. pneumoniae* (D39 strain, ~5 × 10^6^ CFU). IL-12p70 (24, 48 hpi) were measured by ELISA (n = 3-4 mice per group). Data are representative of two independent experiments. **(C)** Flow cytometry analysis of IL-12p70 in lung immune cells from PBS or *S. p* (~5 × 10^6^ CFU) infected C57BL/6J mice at 24 hpi (n = 3 mice per group). Data are representative of three independent experiments. **(D)** Total cell numbers of IL-12p70^+^ lung immune cells from **(C)** were enumerated. **(E, F)** CCR2^−/−^ and WT littermates mice were infected (i.n.) with *S. pneumoniae* as in **(A)** Total cell numbers of lung Ly6C^hi^ monocyte **(E)** and neutrophils **(F)** were enumerated (n = 3–4 mice per group). Data are representative of three independent experiments. **(G)** CCR2^−/−^ and WT littermate mice were infected with *S. pneunoniae* as in panel **(A)** IL-12p70 in lung homogenates (24 hpi) were measured by ELISA (n = 3 mice per group). Data are representative of two independent experiments. Graphs represent the mean with error bars indicating SEM. *p*-values determined by one-way ANOVA Tukey’s multiple comparison test. Significance is represented by asterisk, where *p < 0.05, **p < 0.001, ***p < 0.0001.

Interestingly, *S. pneumoniae*-induced lung IL-12p70 was lost in STING^−/−^ mice (**Figure 1A**). *Streptococcus pneumoniae* secretes cyclic di-AMP that is a STING ligand. STING can also be activated *via* cGAS that senses cytosolic DNA (17–19). We found that cGAS^−/−^ mice failed to make lung IL-12p70, suggesting that *S. pneumoniae*-induced lung IL-12p70 was likely induced by DNA, not cyclic di-AMP (**Figure 1B**). As a control, *S. pneumoniae* induced lung TNFα was unaltered in the cGAS^−/−^ mice (**Figure S1K**).

We wanted to determine the cellular source of IL-12p70 during *S. pneumonia* infection by intracellular cytokine stain. Monocyte, monocyte-derived DCs (moDCs), and monocyte-derived macrophage (moMACs) produced IL-12p70 in the lung during pneumococcal infection, while lung conventional DCs (cDC), T cells, or NK cells did not produce IL-12p70 (**Figures 1C, D**). CCR2 binds to MCP-1 mediating monocyte migration (20). We found that CCR2^−/−^ mice failed to recruit Ly6C^hi^ monocyte (CD11b^+^Ly6C^hi^) into the lung during pneumococcal pulmonary infection (**Figure 1E**). In comparison, the CCR2^−/−^ mice had unaltered lung neutrophils infiltration during the pneumococcal infection (**Figure 1F**). We then examined IL-12p70 production in CCR2^−/−^ mice. CCR2^−/−^ mice failed to make lung IL-12p70 upon *S. pneumoniae* infection (**Figure 1G**). Together, the data suggested that cGAS–STING promoted monocyte production of lung IL-12p70 during *S. pneumoniae* infection.

### Two Waves of Lung IFNγ Production During *S. pneumoniae* Infection

**Figure 2:**
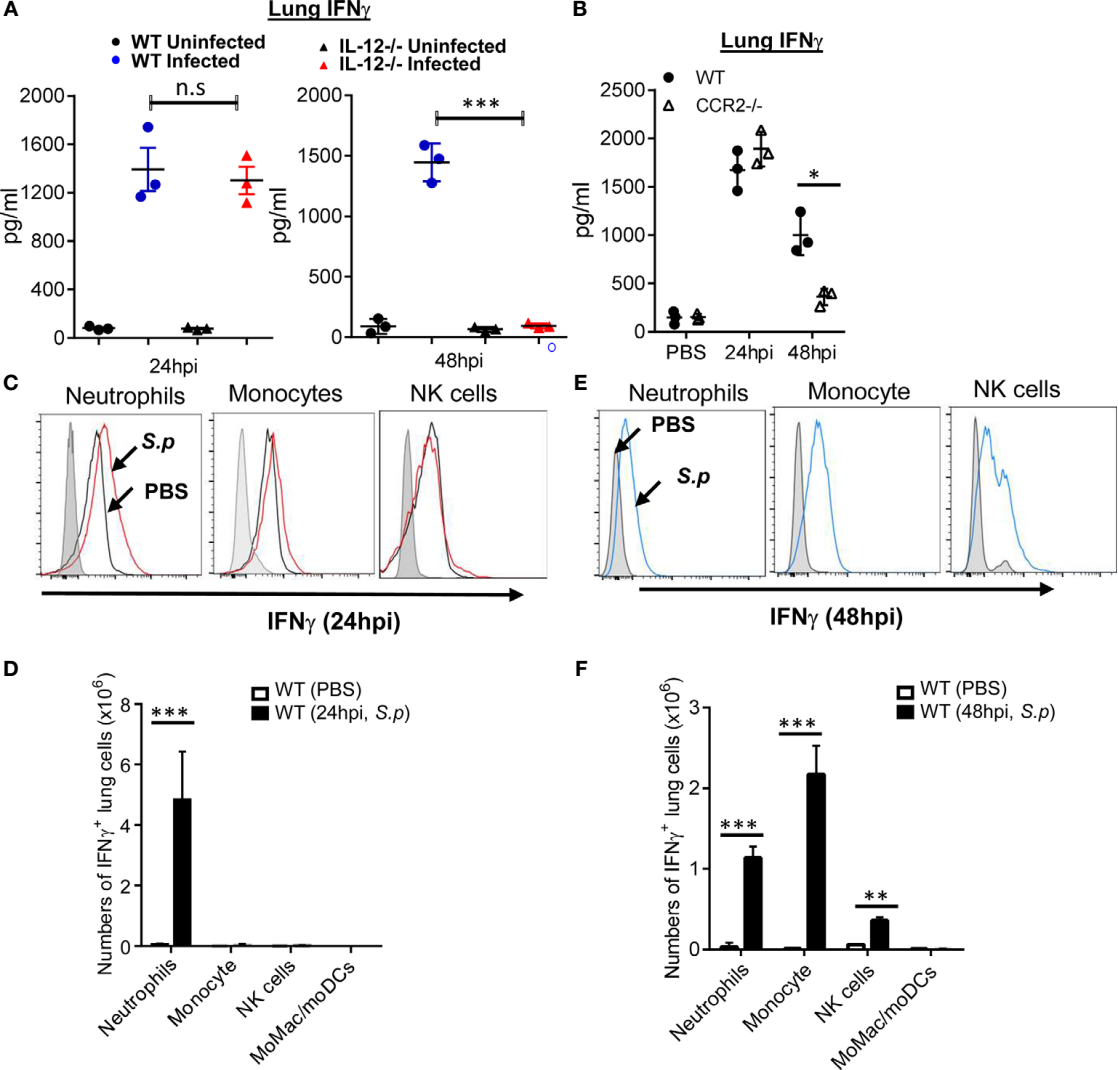
Monocyte and IL-12p70 promote *S. pneumoniae*-induced late-stage lung IFNγ production. **(A)** IL-12p70^−/−^ and WT littermates mice were infected (i.n.) with *S. pneumoniae* (D39 strain, ~5 × 10^6^ CFU). IFNγ in lung homogenates were measured at 24 and 48 hpi by ELISA (n = 3 mice per group). Data are representative of two independent experiments. **(B)** CCR2^−/−^ and WT littermates mice were infected (i.n.) with *S. pneumoniae* as in **(A)**. IFNγ in lung homogenates were measured at 24 and 48 hpi by ELISA (n = 3–4 mice per group). Data are representative of three independent experiments. **(C, E)** Flow cytometry analysis of YFP expression (IFNγ) in lung immune cells from PBS or *S. p* (~5 × 10^6^ CFU) infected IFNγ reporter mice at 24 hpi **(C)** and 48 hpi **(E)** (n = 3–4 mice per group). Data are representative of three independent experiments. **(D, F)** Total cell numbers of IFNγ^+^ lung immune cells in **(C, E)** were enumerated. Graphs represent the mean with error bars indicating SEM. *p*-values determined by one-way ANOVA Tukey’s multiple comparison test. Significance is represented by asterisk, where *p < 0.05, **p < 0.001, ***p < 0.0001, n.s., not significant.

IL-12p70 drives IFNγ production (21, 22). *Streptococcus pneumoniae* infection induces strong IFNγ production in the lungs (9–12). We found that IL-12p70^−/−^ mice failed to make IFNγ upon *S. pneumoniae* infection at 48 hpi, but not at 24 hpi (**Figure 2A**). We reasoned that there were at least two waves of lung IFNγ production during *S. pneumoniae* infection, and only the second wave of lung IFNγ was dependent on IL-12p70. CCR2^−/−^ mice lack lung IL-12p70 production. Similar to the IL-12p70^−/−^ mice, CCR2^−/−^ mice were defective in the late, but not early *S. pneumoniae*-induced lung IFNγ production (**Figure 2B**).

We hypothesized that different immune cells were responsible for lung IFNγ production during the early and late stages. We used IFNγ-YFP reporter mice to detect lung IFNγ-producing cells. Lung immune cells were analyzed by flow cytometry (**Figures S2A–E**). We found that, at 24 hpi, neutrophils were the predominant lung IFNγ−producing cells (**Figures 2C, D**), which is consistent with a recent report (23). However, by 48 hpi, Ly6C^hi^ monocytes and natural killer (NK) cells also produced lung IFNγ-producing cells (**Figures 2E, F**). Neither CD3^+^ T cells, macrophages (CD11b^+^ CD64^+^ CD11c^+/−^ Ly6C^low^) nor dendritic cells (SiglecF^−^ CD11c^hi^ MHC II^hi^) produced IFNγ at 48 hpi (**Figure S2F**). Thus, neutrophils produce lung IFNγ at 24 hpi, while monocyte, NK cells, and neutrophils generate lung IFNγ at 48 hpi.

### STING Is Required for *S. pneumoniae-*Induced Type I IFN-Independent, Late-Stage Lung IFNγ Production

**Figure 3:**
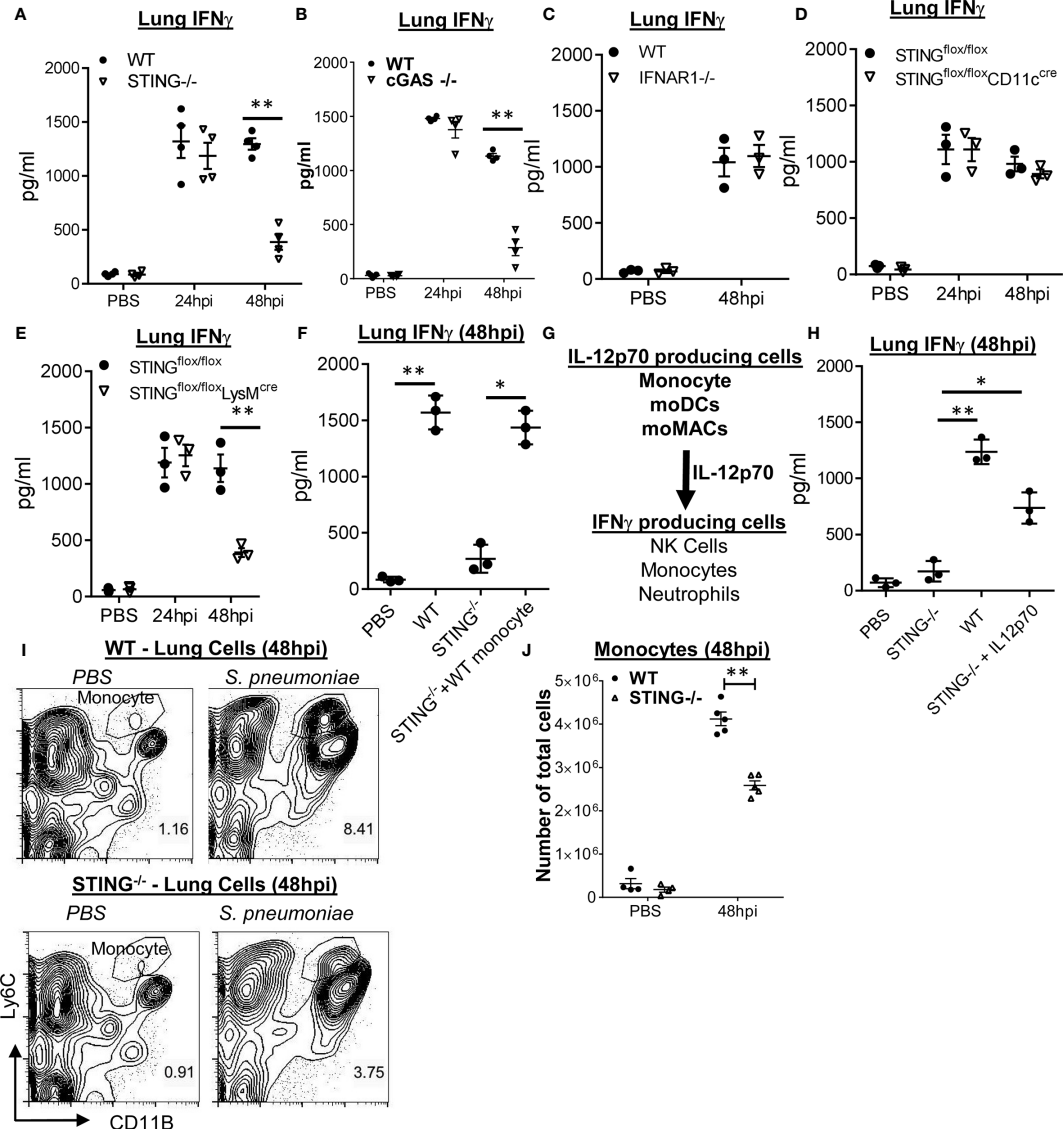
Monocyte expression of STING mediates *S. pneumoniae* induced late-stage lung IFNγ production. **(A, B)** STING^−/−^, cGAS^−/−^, and WT littermates were given PBS or infected (i.n.) with *S. pneumoniae* (D39 strain, ~5 × 10^6^ CFU). IFNγ in lung homogenates (24 and 48hpi) were measured by ELISA (n = 3–4 mice per group). Data are representative of two independent experiments. **(C)** IFNAR1^−/−^ and WT littermate mice were infected with *S. pneunoniae* as in **(A)**. IFNγ in lung homogenates (48 hpi) were measured by ELISA (n = 3 mice per group). Data are representative of two independent experiments. **(D, E)** STING^fl/fl^CD11C^Cre^, STING^fl/fl^LysM^Cre^, and STING^fl/fl^ littermates mice were infected (i.n.) with *S. pneumoniae* as in **(A)**. IFNγ in lung homogenates (24, 48 hpi) were measured by ELISA (n = 3 mice per group). Data are representative of two independent experiments. **(F)** STING^−/−^ and WT littermates were infected with *S. pneumoniae* as in panel **(A)** At 16 hpi, 1 million bone marrow WT Ly6C^hi^ monocytes were adoptively transferred (i.n.) into STING^−/−^ mice (STING^−/−^ + WT monocyte). IFNγ in lung homogenates was measured at 48 hpi by ELISA (n = 3 mice per group). Data were representative of two independent experiments. **(G)** A diagram of lung IFNγ production by monocyte-derived IL-12p70. **(H)** STING^−/−^ and WT littermates were infected with *S. pneumoniae* as in **(A)**. At the 16 hpi, recombinant IL-12p70 (1 µg) was administered (i.n.) into STING^−/−^ mice. IFNγ in lung homogenates was measured at 48 hpi by ELISA (n = 3 mice per group). Data are representative of two independent experiments. **(I, J)** STING^−/−^ and WT littermates mice were infected (i.n.) with *S. pneumoniae* as in **(A)**. Ly6C^hi^ monocytes **(I)** were identified in total lung cells at 48 hpi by flow cytometry. Total cell numbers of lung Ly6C^hi^ monocytes **(J)** were enumerated (n = 3–4 mice per group). Data were representative of three independent experiments. Graphs represent the mean with error bars indicating SEM. *p*-values determined by one-way ANOVA Tukey’s multiple comparison test. Significance is represented by asterisk, where *p < 0.05, **p < 0.001.

STING^−/−^ mice failed to make IL-12p70 during *S. pneumoniae* infection (**Figure 1A**). Similar to the CCR2^−/−^ and IL-12p70^−/−^ mice, we observed a significant reduction in lung IFNγ production in the STING^−/−^ mice at 48 hpi, but not at 24 hpi (**Figure 3A**). cGAS^−/−^ mice also failed to make lung IFNγ at 48 hpi (**Figure 3B**), suggesting that cytosolic sensing of DNA, not *S. pneumoniae* cyclic di-AMP, promoted lung IFNγ. Lastly, IFNAR1^−/−^ mice had unaltered IFNγ production in the lung upon *S. pneumoniae* infection (**Figure 3C**). Thus, cGAS–STING–IL12p70–IFNγ signaling is likely type I IFN independent.

### LysM^cre^STING^fl/fl^ Mice Lack *S. pneumoniae* Induced Late-Stage Lung IFNγ Production

Monocyte/monocyte-derived cells are critical for lung IL-12p70 and the late-stage IFNγ production (**Figures 1** and **2**). We hypothesized that STING expression in monocyte drove lung IFNγ production. We examined *S. pneumonia*-induced lung IFNγ production in CD11c^cre^STING^fl/fl^ and LysM^cre^STING^fl/fl^ mice. CD11c^cre^STING^fl/fl^ mice delete STING gene in alveolar macrophage and dendritic cells (DCs), while LysM^cre^STING^fl/fl^ mice delete STING gene in alveolar macrophage, interstitial macrophage, monocyte/monocyte-derived cells, and neutrophils (24). Notably, neutrophils do not express STING, while NK cells and Ly6C^hi^ monocytes have strong STING expression (13, 14).

We found that LysM^cre^STING^fl/fl^, not the CD11c^cre^-STING^fl/fl^ mice, were defective in the late-stage lung IFNγ production by *S. pneumoniae* infection (**Figures 3D, E**), suggesting that STING expressing in interstitial macrophage, monocyte/monocyte-derived cells, not DCs or alveolar macrophage, was likely responsible for *S.pneumoniae*-induced late-stage lung IFNγ production.

### STING Expression in Ly6C^hi^ Monocyte Promotes *S. pneumoniae-*Induced Lung IFNγ at 48 hpi

To further establish that STING expression in monocyte/monocyte-derived cells is critical for lung IFNγ production, we adoptively transferred (i.n) WT bone marrow Ly6C^hi^ monocyte into STING^−/−^ mice at 16 hpi and determined lung IFNγ production at 48 hpi. We found that STING^−/−^ mice receiving WT Ly6C^hi^ monocyte produced lung IFNγ at 48 hpi (**Figure 3F**). We concluded that STING expression in monocyte/monocyte-derived cells promotes the late-stage lung IFNγ production during *S. pneumoniae* infection.

Besides monocyte, neutrophils and NK cells produce lung IFNγ at 48 hpi (**Figure 2F**). We proposed that STING expression in monocyte/monocyte-derived cells produces IL-12p70 that drove late-stage lung IFNγ production by NK cells and neutrophils during pneumococcal infection (**Figure 3G**). Indeed, intranasal administration of recombinant IL-12p70 at 16 hpi restored IFNγ production in STING^−/−^ mice (**Figure 3H**).

We also examined lung monocyte infiltration in STING^−/−^ mice during *S. pneumoniae* infection. We observed a mild decrease in lung Ly6C^hi^ monocytes in STING^−/−^ mice at 48 hpi (**Figures 3I, J**). Furthermore, STING^−/−^ had similar *S. pneumoniae*-induced MCP-1 production as the WT mice (S1D). We, thus, preferred the hypothesis that STING expression in monocyte senses DNA and drives IL-12p70 production to promote late-stage lung IFNγ production.

### Activation of the STING Pathway by DNA Is not Sufficient to Induce IL-12p70 and IFNγ in Ly6C^hi^ Monocyte

cGAS–STING pathway senses cytosolic DNA from invading pathogens or self-DNA by damaged host cells. We examined if monocyte/monocyte-derived cells were directly infected by *S. pneumoniae* at 48 hpi, thus may contain cytosolic pathogen DNA. We infected (i.n.) mice with BacLight Green-stained *S. pneumoniae*. At 48 hpi, we examined BacLight Green+ cells in the lung. Neutrophils were heavily infected with *S. pneumoniae* (**Figure S3A**). Ly6C^hi^ monocyte, NK cells, or moMAC, however, contained few labeled bacteria (**Figures S3B–D**), indicating that the *S. pneumoniae* may not directly release pathogen DNA into the cytosol of these cells.

**Figure 4:**
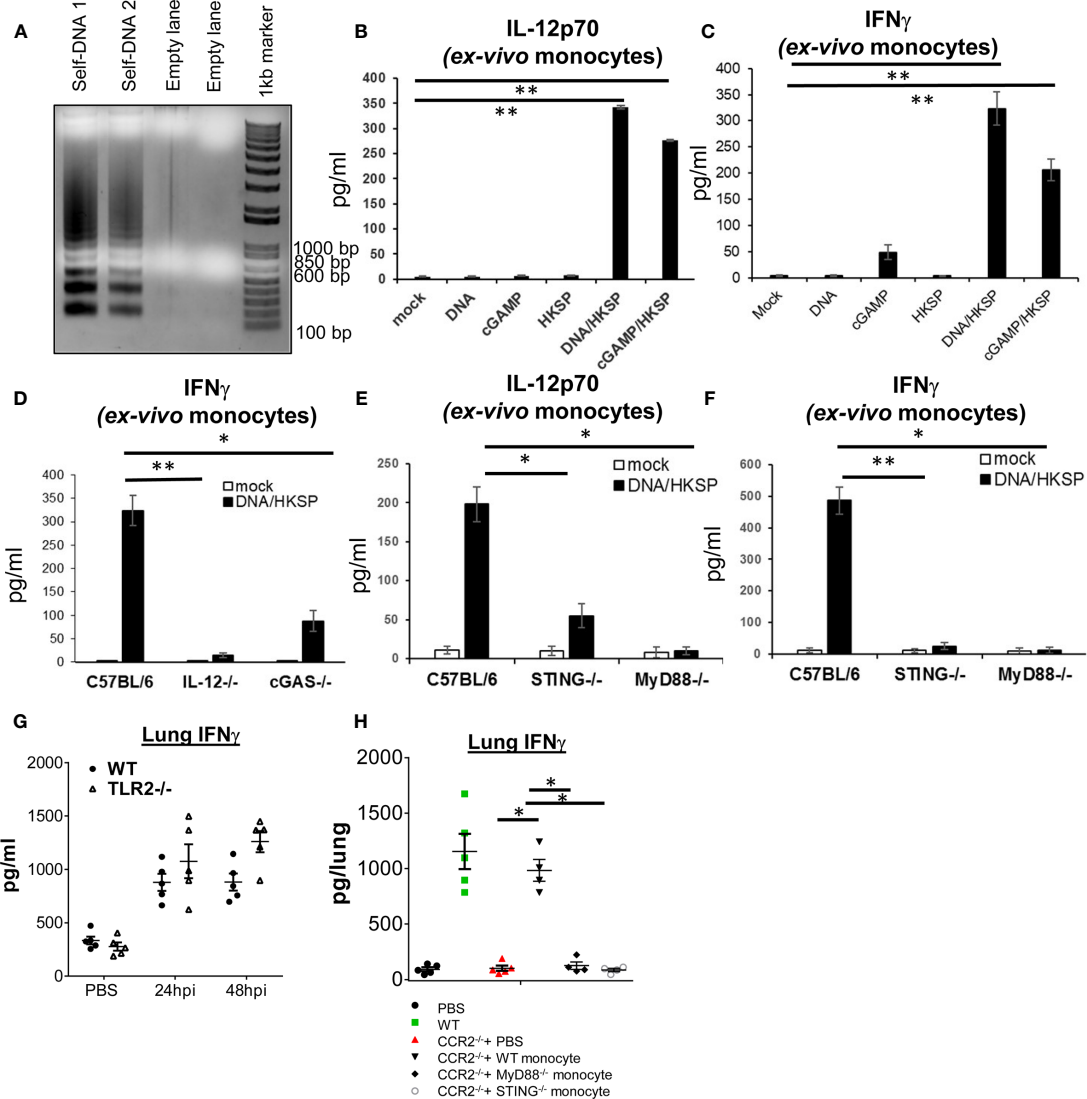
Ly6C^hi^ monocyte production of IL-12p70 and IFNγ require the activation of both cGAS–STING and MyD88 pathways. **(A)** Splenocytes isolated from a C57BL/6J mouse were cultured *ex vivo* for 4 days. Genomic DNA was extracted and run on an agarose gel (Self DNA 1 and Self DNA2). **(B, C)** Ly6C^hi^ monocyte isolated from C57BL/6J mice were activated with self-DNA (1.5 µg/ml), HKSP (5 × 10^6^ CFU/ml), 2′3′-cGAMP (4 µg/ml) or DNA + HKSP, HKSP + 2′3′-cGAMP for 17 h IL-12p70 **(B)** and IFNγ **(C)** were measured in the culture supernatant by ELISA. Data are representative of three independent experiments. **(D–F)** Ly6C^hi^ monocyte isolated from indicated mice were activated with self-DNA (1.5 µg/ml) plus HKSP (5 × 10^6^ CFU/ml) for 17 h as in **(B)**. IFNγ and IL-12p70 were measured in the culture supernatant by ELISA. Data are representative of three independent experiments. **(G)** TLR2^−/−^ and their WT littermates were infected (i.n.) with PBS or *S. p* (D39 strain, ~5 × 10^6^ CFU). IFNγ in lung homogenates was measured by ELISA at 24 and 48 hpi (n = 4–5 mice/group). Data are representative of two independent experiments. **(H)** CCR2^−/−^ and WT littermates were infected with *S. pneumoniae* (D39 strain, ~8 × 10^6^ CFU). At the 16 hpi, 1 million bone marrow WT, MyD88^−/−^, or STING^−/−^ Ly6C^hi^ monocytes were adoptively transfer (i.n.) into CCR2^−/−^ mice. IFNγ in lung homogenates were measured at 48 hpi by ELISA (n = 4–5 mice/group). Data are representative of two independent experiments. Graphs represent the mean with error bars indicating SEM. *p*-values determined by one-way ANOVA Tukey’s multiple comparison test. Significance is represented by asterisk, where *p < 0.05, **p < 0.001.

During live infection, besides live bacteria and bacteria components, there were dead host cells in the lung that could release their DNA and activate the cGAS–STING pathway. We activated monocyte with mouse genomic DNA isolated from apoptotic mouse splenocytes (**Figure 4A**). We isolated Ly6C^hi^ monocytes from the bone marrow and stimulated them with mouse genomic apoptotic DNA. Surprisingly, we found that the apoptotic DNA alone did not generate IL-12p70 or IFNγ in the isolated Ly6C^hi^ monocyte (**Figures 4B, C**). We also used cGAMP to activate the Ly6C^hi^ monocyte. Again, cGAMP did not induce IL-12p70 (**Figure 4B**). As control, both DNA and cGAMP activated Ly6C^hi^ monocytes to produce TNFα and IFNβ (**Figures S4A, B**). Thus, DNA sensing alone cannot activate Ly6C^hi^ monocyte to produce IL-12p70 or IFNγ.

### Heat-Killed *Streptococcus pneumoniae* and Apoptotic DNA Together Induce IL-12p70 and IFNγ in Ly6C^hi^ Monocyte

During live *S. pneumoniae* infection, monocyte and monocyte-derived cells likely encounter both PAMP, e.g., TLR agonists, and DAMP, e.g., host DNA released from dead cells. We hypothesized that the cGAS–STING pathway may synergize with the TLR pathway to induce IL-12p70 and IFNγ in Ly6C^hi^ monocyte. We activated monocyte with HKSP plus apoptotic DNA. Indeed, HKSP/DNA stimulation induced IL-12p70 and IFNγ in monocyte (**Figures 4B, C**). Similarly, HKSP/cGAMP induced IL-12p70 and IFNγ (**Figures 4B, C**). HKSP alone did not induce IL-12p70 or IFNγ in the monocyte (**Figures 4B, C**). We concluded that the induction of IL-12p70-IFNγ in monocyte required the synergistic activation of DNA and TLRs signaling.

### MyD88 Is Required for HKSP/DNA Induced IL-12p70 and IFNγ Production in Ly6C^hi^ Monocyte

As expected, HKSP/DNA did not stimulate IFNγ production in Ly6C^hi^ monocyte from IL-12p70^−/−^ mice (**Figure 4D**). cGAS^−/−^ and STING^−/−^ monocyte were also defective in IFNγ production by HKSP/DNA (**Figures 4D–F**) confirming that the cGAS–STING pathway was required. As control, HKSP activated TNFα in IL-12p70^−/−^ and cGAS^−/−^ monocyte (**Figure S4C**). HKSP activated the TLR2–MyD88 pathway (25). We found that Ly6C^hi^ monocyte from MyD88^−/−^ mice failed to make IL-12p70 or IFNγ (**Figures 4E, F**), suggesting that monocyte production of IL-12p70 by HKSP/DNA requires the MyD88 pathway. As a control, MyD88^−/−^ monocyte made IFNβ and TNFα in response to HKSP/DNA (**Figures S4D, E**).

To ask if TLR2 is required for lung IFNγ production, we infected TLR2^−/−^ mice with *S. pneumoniae*. Unlike the STING^−/−^ or cGAS^−/−^ mice, the lung IFNγ production was similar in WT and TLR2^−/−^ mice (**Figure 4G**). We suspected that additional TLRs pathways, such as TLR9 (26), might synergize with the cGAS–STING pathway for lung IFNγ production during pathogen infection, compensating for the loss of TLR2.

To establish that MyD88 are required for *S. pneumoniae* induced lung IFNγ, we adoptively transferred (i.n.) WT, STING^−/−^, or MyD88^−/−^ monocytes to CCR2^−/−^ mice and examined the IFNγ production in the lung by *S. pneumonia*. Unlike the WT monocyte, neither STING^−/−^ nor MyD88^−/−^ monocyte restored lung IFNγ production in the CCR2^−/−^ mice (**Figure 4H**). Thus, both MyD88 and STING expression in the monocyte are required for lung IFNγ production *in vivo*.

## Discussion

In this report, we showed that STING synergizes with MyD88 to induce IL-12p70 and IFNγ in the lung. STING is particularly required for the late-stage (48 hpi) lung IFNγ production during *S. pneumoniae* infection. This unique requirement likely reflects the need for IL-12p70 production since the initial lung IFNγ production by *S. pneumoniae* is IL-12p70 independent (23).

Previously, Temizoz et al. showed that cGAMP, in combination with CpG ODN, stimulated IFNγ production in PBMCs (26). Furthermore, cGAMP and CpG ODN together, acting as an antigen-free anticancer agent, reduced tumor size significantly compared to cGAMP alone in the EG-7 and B16-F10 mouse tumor models (26). They further showed that IL-12p70 was required for the synergistic induction of IFNγ in PBMCs (26). Different from ours, Temizoz et al. showed that type I IFN was needed for the IFNγ production (26). Type I IFN is required for IL-18 production in moMACs (27). IL-18, also known as IFNγ-inducing factor, can induce IFNγ production (21, 22, 28). In our experimental setting, lung production of IFNγ does not require type I IFN. Nevertheless, it is likely that that type I IFN, *via* the production of IL-18, together with IL-12p70, could further augment IFNγ production.

It has long been known that DCs production of IL-12p70 requires at least two stimuli (29–33). This dual requirement is likely a safeguard to avoid the possible detrimental effects of uncontrolled IL-12p70-medicated Th1 responses. Napolitani et al. showed that in both human and mouse DCs, TLR3 and TLR4 potently synergized with TLR7, TLR8, and TLR9 to induce IL-12p70 and IL-23, leading to enhanced and sustained Th1 responses (29). Here, we found that STING-mediated cytosolic DNA sensing pathway synergize with TLR2 pathway in monocyte for IL-12p70 and IFNγ production. Nevertheless, it is likely that STING pathway can synergize with other PRRs for IL-12p70 and IFNγ production because TLR2^−/−^ mice did not have defect in lung IFNγ production during *S. pneumoniae* infection. How STING and TLRs synergistically induce IL-12p70 is unclear. TLRs activation takes place on the plasma membrane or endosome, while STING activation happens at the ER–Golgi interface. It is tempting to speculate that a spatiotemporal activation of STING and TLRs may aid in IL-12p70 production.

The discovery of two waves of lung IFNγ production during *S. pneumoniae* infection may clarify the role of IFNγ in pneumococcal infection. Using IFNγ^−/−^ mice or anti-IFNγ neutralizing Ab, previous studies were inconclusive (9–12). We speculated that the two waves of lung IFNγ may play opposite roles in host defense against pneumococcal infection. The neutrophils-mediated, IL-12p70-independent early lung IFNγ may be beneficial by generating M1 macrophages to neutralize bacteria. The late-stage lung IFNγ in pneumococcal infection, however, may be detrimental because in patients with *S. pneumoniae* sepsis, IFNγ was elevated and correlated with increased mortality (7). We speculated that persistent IFNγ production may promote sustained inflammation that may enhance tissue damage and mortality.

Ly6C^hi^ monocyte has emerged as a key player in pathogen-induced IFNγ production in the mucosal surface (34, 35). Ly6C^hi^ monocytes are rapidly recruited to sites of infection and differentiate into macrophages and dendritic cells. Two recent studies found that CCR2^−/−^ mice, which lack infiltrating Ly6C^hi^ monocyte, produced significantly less IFNγ in the lung during pulmonary *Legionella pneumophila* infection (34, 35). Similar to our finding, they found that IL-12p70 is required for IFNγ production and identified infiltrating monocyte as the major source of IL-12p70 (34, 35). Another study found that during vaginal HSV-2 infection, Ly6C^hi^ monocytes produce IL-18, which activates NK cells to produce IFNγ (36). Thus, a new paradigm emerges that during mucosal pathogen infection, infiltrating Ly6C^hi^ monocyte produces IL-12p70 or IL-18 that instructs NK cells or T cells to produce IFNγ.

In summary, the activation of the STING pathway in monocyte/monocyte-derived cells can synergize with the MyD88 pathway to drive IFNγ production during pneumococcal infection that may influence the development of adaptive immunity.

## Data Availability Statement

The raw data supporting the conclusions of this article will be made available by the authors, without undue reservation.

## Ethics Statement

All experiments with mice were performed by the regulations and approval of the Institutional Animal Care and Use Committee from the University of Florida (protocol number 201909362).

## Author Contributions

HRT, SP, and LJ conceived the research. LJ designed the experiments, wrote the manuscript, and supervised the research. SP, HT, HG, SM, and LJ performed experiments and analyzed the data. SP drafted the manuscript. All authors contributed to the article and approved the submitted version.

## Funding

This work was supported by NIH grants AI110606, AI125999, AI132865, and HL152163 (to LJ). SM was supported through The American Association of Immunologists Careers in Immunology Fellowship Program.

## Conflict of Interest

The authors declare that the research was conducted in the absence of any commercial or financial relationships that could be construed as a potential conflict of interest.

## Publisher’s Note

All claims expressed in this article are solely those of the authors and do not necessarily represent those of their affiliated organizations, or those of the publisher, the editors and the reviewers. Any product that may be evaluated in this article, or claim that may be made by its manufacturer, is not guaranteed or endorsed by the publisher.

## Supplementary Material

The Supplementary Material contains four figures and one table and can be found online.
