# Supplementary material for: cGAS–STING and MyD88 Pathways Synergize in Ly6Chi Monocyte to Promote Streptococcus pneumoniae-Induced Late-Stage Lung IFNγ Production
Source: Front Immunol. 2021 Aug 26;12:699702. doi: 10.3389/fimmu.2021.699702 (PMC8427188; doi:10.3389/fimmu.2021.699702)
Supplement: Supplementary file 1 [file DataSheet_1.pdf]

Supplementary Material

1 Supplementary Figures and Tables

1.1 Supplementary Table

Supplemental Table 1: Key resources

REAGENT or RESOURCE	IDENTIFIER	SOURCE
S. pneumonia D39 serotype 2		ATCC, Manassas, VA, USA
Tryptic soy broth	T8907	Sigma Aldrich, St. Louis, MO, USA
Phosphate buffer saline	K812	Amresco, Solon, OH, USA
BacLight Green	B35000	ThermoFisher Scientific, Grand Island, NY, USA
Tissue protein extraction reagent	78510	ThermoFisher Scientific, Grand Island, NY, USA
Protease inhibitor	11836153001	Roche, Indianapolis, IN, USA
IL- 1β ELISA kit	88-7013	eBiosciences, San Diego, CA, USA
IL- 6 ELISA kit	88- 7064	eBiosciences, San Diego, CA, USA
IL- 12p70 ELISA kit	88- 7921	eBiosciences, San Diego, CA, USA
TNF- α ELISA kit	88- 7324	eBiosciences, San Diego, CA, USA
MCP- 1 ELISA kit	88- 7391	eBiosciences, San Diego, CA, USA
IFN- γ ELISA kit	88- 7314	eBiosciences, San Diego, CA, USA
IFN- β ELISA kit	42410- 1	PBI Interferon Source, Piscataway, NJ, USA
DNase I	10104159001	Roche, Mannheim, Germany
Liberase TM	05401119001	Roche, Mannheim, Germany
ACK lysis buffer	A1-0492-01	Gibco, Grand island, NY, USA
Cytofix/CytopermTM kit	555028	BD Biosciences, Franklin Lakes, NJ, USA
Golgi-plug	555029	BD Bioscience, Franklin Lakes, NJ, USA
Permeabilization buffer	554723	BD Bioscience, Franklin Lakes, NJ, USA
EasySep Mouse MonocyteIsolation kit	19861A	STEMCELL Technologies, Vancouver, Canada
RPMI	11965118	Invitrogen, Waltham, MA, USA
Fetal bovine serum	35-011-CV	Corning, Manassas, VA, USA
L- Glutamine	25-005-CI	Corning, Manassas, VA, USA
Sodium pyruvate	11360- 070	Gibco, Grand island, NY, USA
HEPES	25- 060-CI	Corning, Manassas, VA, USA
Non- essential amino acids	11140- 050	Gibco, Grand island, NY, USA
2- mercaptoethanol	M6250	Sigma Aldrich, St. Louis, MO, USA.
Penicillin/ streptomycin	30-002-CI	Corning, Manassas, VA, USA

Heat killed *Streptococcus pneumoniae*	tlrl-hksp	Invivogen, SanDiego, CA, USA
DNeasy Blood & Tissue Kit	69504	Qiagen, Hilden, Germany
Anti-Ly6C antibody	HK1.4	Biolegend, San Diego, CA, USA
Anti-CD11b antibody	M1/70	Biolegend, San Diego, CA, USA
Anti-Ly6G antibody	1A8	Biolegend, San Diego, CA, USA
Anti-CD11c antibody	N418	Biolegend, San Diego, CA, USA
Anti-NK1.1 antibody	PK136	Biolegend, San Diego, CA, USA
Anti-MHC II antibody	M5/114.15.2	Biolegend, San Diego, CA, USA
Anti-CD103 antibody	2E7	Biolegend, San Diego, CA, USA
Anti-CD3 antibody	145.2C11	Biolegend, San Diego, CA, USA
Anti-CD64 antibody	X54-5/7.1	Biolegend, San Diego, CA, USA
Anti-SiglecF antibody	S17007L	Biolegend, San Diego, CA, USA
Anti-IFN γ antibody	XMG1.2	Biolegend, San Diego, CA, USA
Anti-IL-12p35 antibody	27537	Thermofisher Scientific, Grand Island, NY, USA
STING$^{-/-}$ mouse		Made by Dr. Jin
STING$^{fl/fl}$ mouse		Made by D. Jin
CCR2$^{-/-}$ mouse	004999	Jackson laboratory
cGAS$^{-/-}$ mouse	026554	Jackson laboratory
IL-12p70$^{-/-}$ mouse	002692	Jackson laboratory
IFNAR1$^{-/-}$ mouse	32045	Jackson laboratory
MyD88$^{-/-}$ mouse	009088	Jackson laboratory
TLR2$^{-/-}$ mouse		Jackson laboratory
IFN-γ YFP reporter mouse	017581	Jackson laboratory

1.2 Supplementary Figures

Supplementary Figure 1.

Fig S1: The innate responses to S. pneumoniae in lungs are largely intact in STING$^{-/-}$ mice

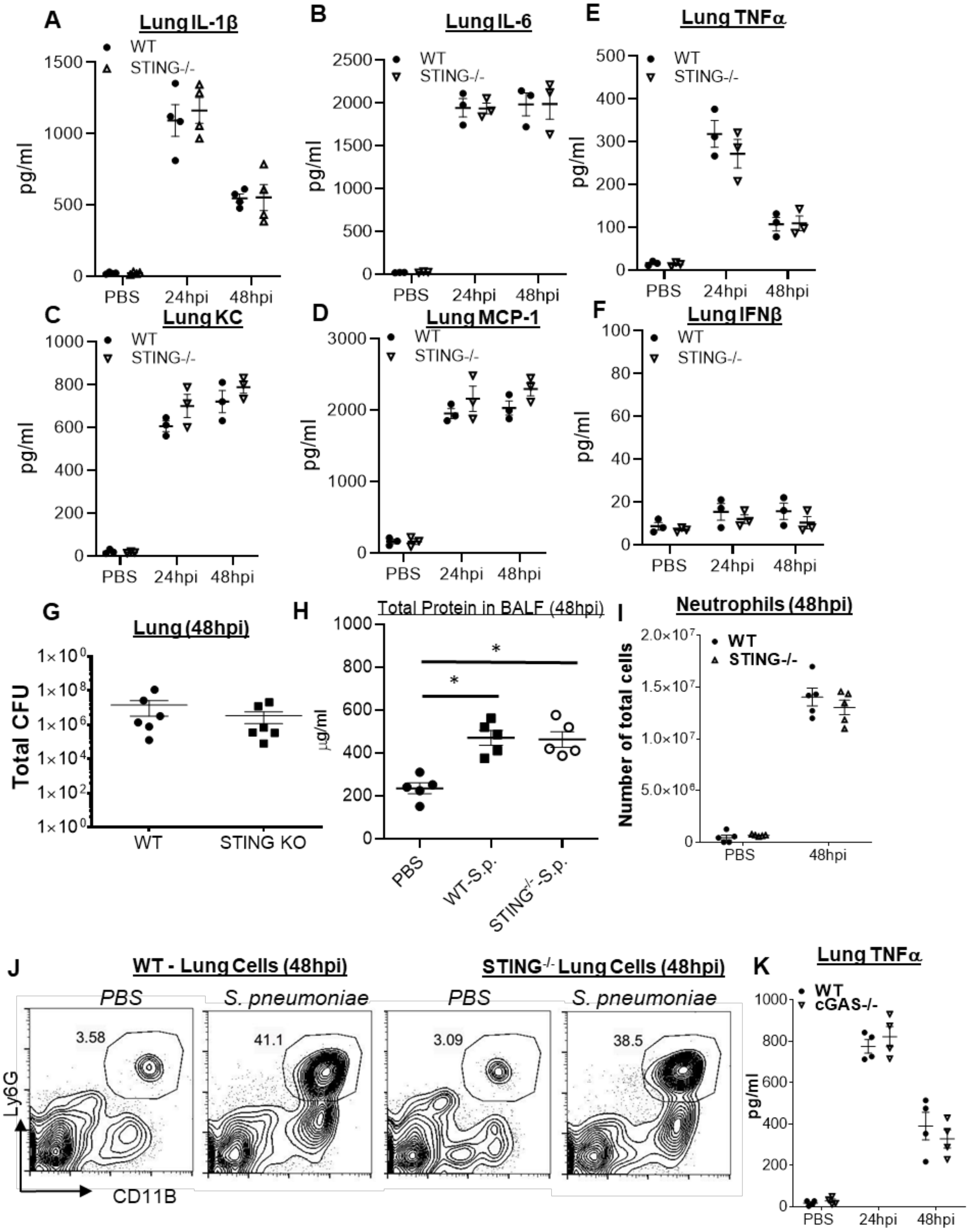

Figure S1. **The innate responses to *S. pneumoniae* infection are largely intact in STING$^{-/-}$ mice.** **A-F.** STING$^{-/-}$ and WT littermates mice were infected (i.n.) with *S.pneumoniae* (D39 strain, ~5x10^6 c.f.u.). Indicated cytokines and chemokines in lung homogenates (24hpi, 48hpi) were measured by ELISA. (n=3-4 mice per group). Data are representative of 2 independent experiments. **G-H.** STING$^{-/-}$ and WT littermates mice were infected (i.n.) with *S.pneumoniae* as in **A**. Bacterial burden was determined in the lungs at 48hpi (**H**). Total protein in BALF was quantified (**I**). (n=5-6 mice per group). Data are representative of 2 independent experiments. **I-J.** STING$^{-/-}$ and WT littermates mice were infected (i.n.) with *S.pneumoniae* as in **A**. Lung neutrophil (**J**) were identified by flow cytometry at 48hpi. Total cell numbers of lung neutrophils (**I**) were enumerated. (n=5 mice per group). Data were representative of 3 independent experiments. **K.** cGAS$^{-/-}$ and WT littermates mice were infected (i.n.) with *S.pneumoniae* (D39 strain, ~5x10^6 c.f.u.). TNFα in lung homogenates (24hpi, 48hpi) were measured by ELISA. (n=3-4 mice per group). Data are representative of 2 independent experiments. Graphs represent the mean with error bars indication s.e.m. *P* values determined by one-way ANOVA Tukey's multiple comparison test. Significance is represented by an asterisk, where *=$p < 0.05$.

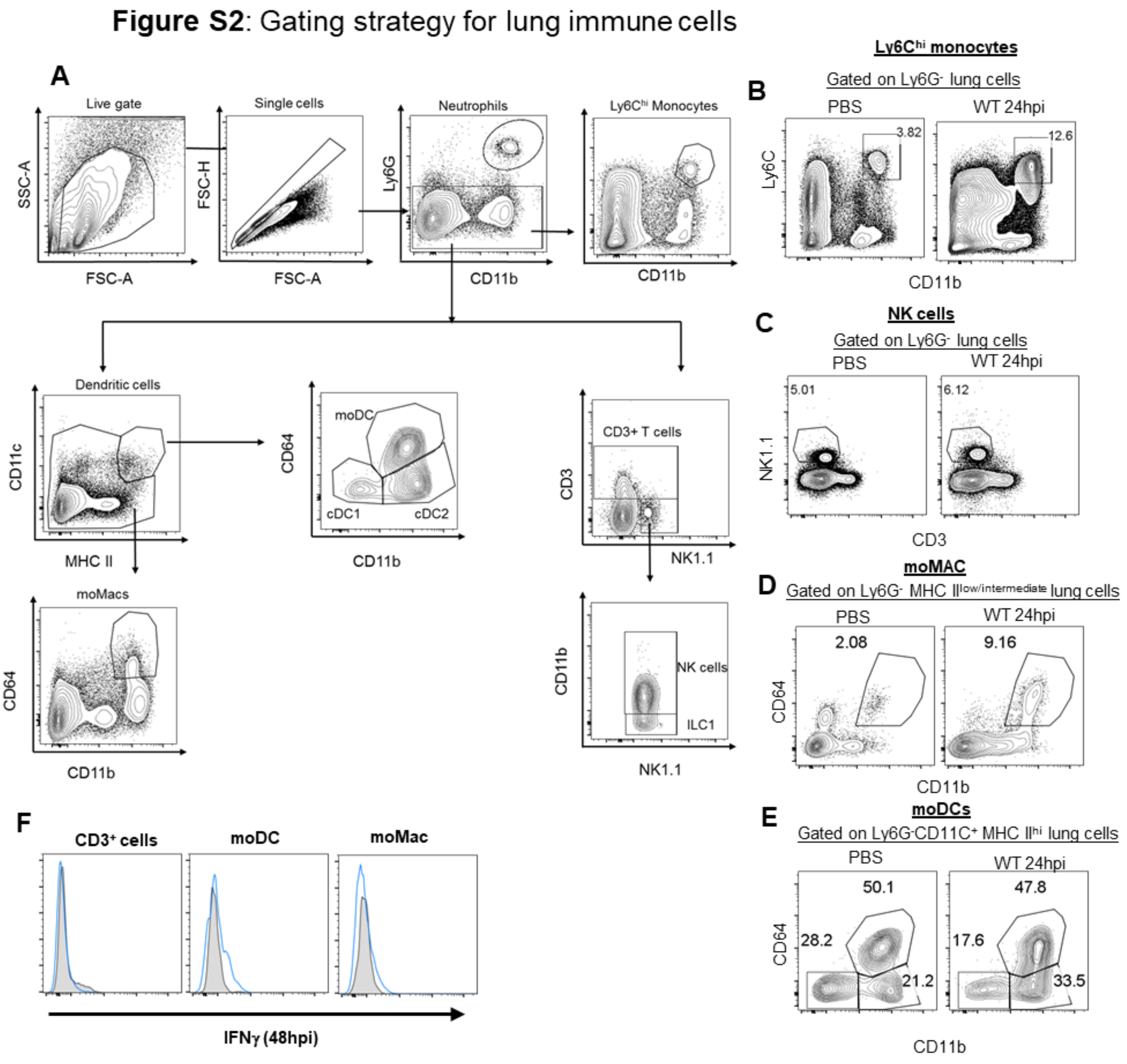

Figure S2. Gating strategy for lung immune cells. A. Flow cytometry-gating strategy used for lung immune cell populations. **B-E**. Flow cytometry analysis of lung immune cells in C57BL/6J mice infected with PBS or *S.p* (~5x10^6 c.f.u.) at 24hpi. **F.** Flow cytometry analysis of YFP expression (IFNγ) in lung immune cells from PBS or *S.p* (~5x10^6 c.f.u.) infected IFNγ reporter mice at 48hpi. (n=3-4 mice per group). Data were representative of 3 independent experiments.

Figure S3: Neutrophils, not, monocyte, are the major *S. pneumoniae* infected lung cells

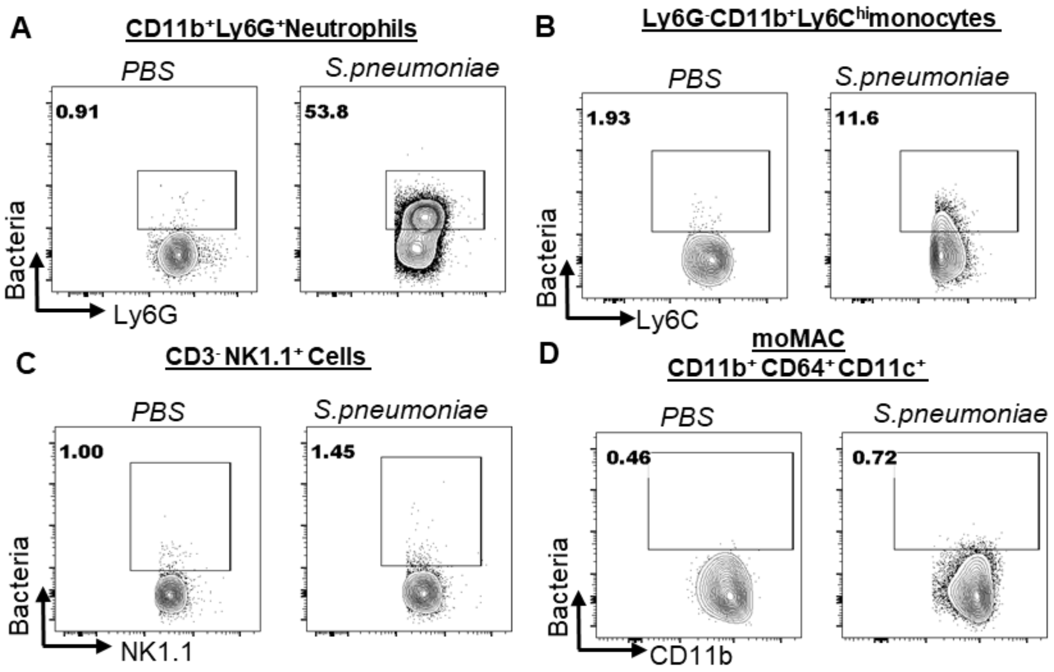

Figure S3. Neutrophils, not monocyte, are the major *S. pneumoniae* infected lung cells at 48hpi. A-D. C57BL/6 mice were given PBS or infected (i.n) with BacLight green stained *S.p* (D39 strain, ~8x10^6c.f.u.). Lung neutrophil, Ly6C^{hi} monocyte, NK cell population, and moMacs were determined and examined for BacLight green expression at 48hpi. (n=3 mice per group). Data were representative of 3 independent experiments.

Figure S4: MyD88 and STING pathways synergistically induce IL-12p70 and IFNγ production in Ly6C[hi] monocyte.

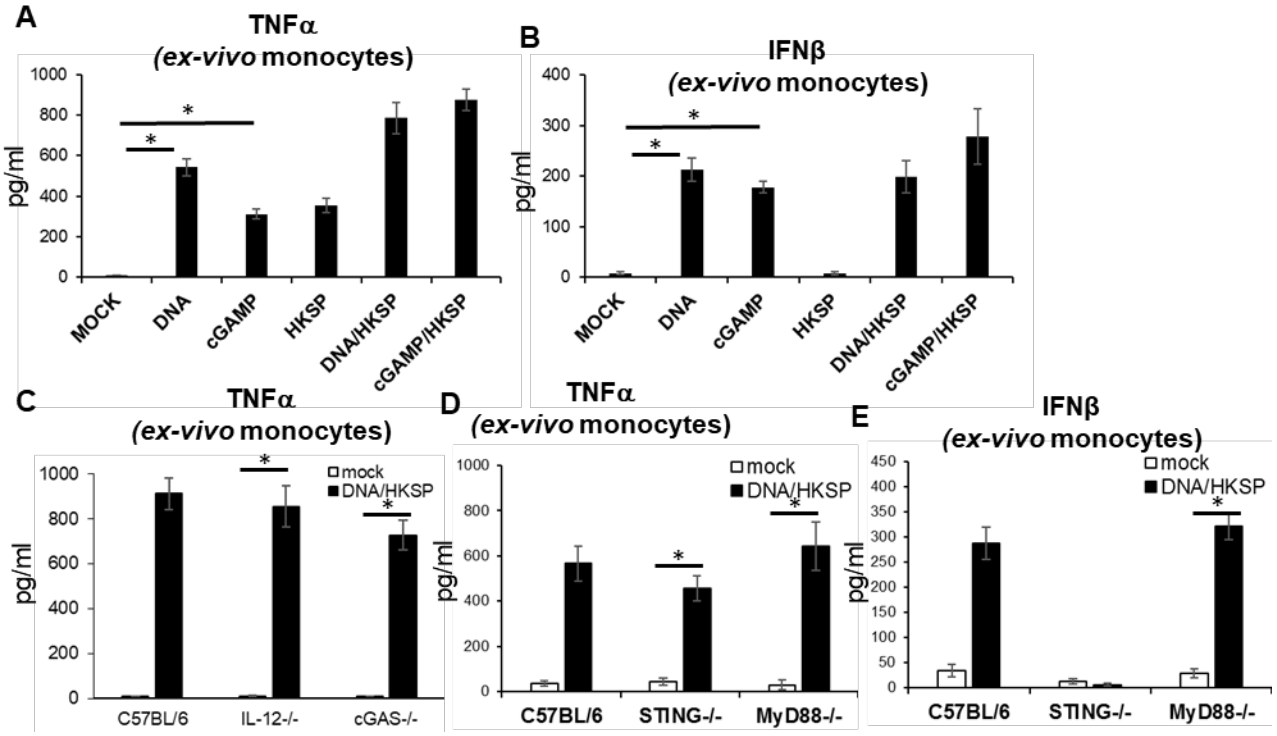

Figure S4. MyD88 and STING pathways synergistically induce IL-12p70 and IFNγ production by Ly6C[hi] monocyte. A-B Ly6C[hi] monocyte isolated from C57BL/6J mice were activated with self-DNA (1.5µg/ml), HKSP ($5x10^6$c.f.u/ml), 2'3'-cGAMP (4µg/ml) or DNA+HKSP, HKSP+ 2'3'-cGAMP for 17hrs. TNFα and IFNβ were measured in the culture supernatant by ELISA. Data were representative of 3 independent experiments. **C-E**. Ly6C[hi] monocyte isolated from indicated mice were activated with self-DNA (1.5µg/ml) plus HKSP ($5x10^6$c.f.u/ml) for 17hrs as in **A**. TNFα and IFNβ were measured in the culture supernatant by ELISA. Data were representative of 3 independent experiments. Graphs represent the mean with error bars indication s.e.m. *P* values determined by one-way ANOVA Tukey's multiple comparison test. Significance is represented by an asterisk, where *=p < 0.05.
